# A Rare Case of an Asymmetric Overgrowth Syndrome in a Kenyan African Child: A Case Report and Review of Literature

**DOI:** 10.7759/cureus.29761

**Published:** 2022-09-29

**Authors:** Diana A Okello, Joseph Mutio, Mary A Masiga, Symon Guthua, Nyambura Kariuki, Catherine Mutinda, Krishan Sarna, Ruth Wanjohi

**Affiliations:** 1 Department of Pedodontics and Preventive Dentistry, University of Nairobi, Nairobi, KEN; 2 Department of Oral and Maxillofacial Surgery, Oral Pathology and Oral Medicine, Division of Oral and Maxillofacial Surgery, University of Nairobi, Nairobi, KEN; 3 Department of Paediatrics and Child Health, University of Nairobi, Nairobi, KEN; 4 Department of Genetics, University of Nairobi, Nairobi, KEN; 5 Department of Radiology, The Nairobi Hospital, Nairobi, KEN

**Keywords:** assymetric overgrowth, akt1, overgrowth syndrome, hemihypertrophy, proteus syndrome

## Abstract

Body overgrowth may be generalized or may affect certain areas. We present a seven-year-old African male with progressive, asymmetric, postnatal overgrowth of the left side of his face and left upper limb. The impression of proteus syndrome (PS) was made based on established clinical diagnostic criteria presented in the literature. Our objective is to highlight the diagnosis based on clinical features, investigations, and a multidisciplinary approach to the management of proteus syndrome.

## Introduction

Overgrowth is described as the symmetrical or asymmetrical increase in body size which may be due to over-nutrition, endocrine conditions, or genetic variations [1,2]. A syndrome may be defined as a discernable complex of traits that occur together and indicate a condition with a definitive cause [3]. Neylon et al. proposed a classification of overgrowth syndromes as those identified in the neonatal period, such as Klippel-Trenaunay-Weber syndrome, CLOVES (congenital lipomatous overgrowth, progressive, complex and mixed truncal vascular malformations, and epidermal nevi), Beckwith-Wiedemann syndrome, and those identified in childhood, such as Proteus syndrome (PS), neurofibromatosis type I [4-8].

PS is an exceedingly rare, sporadic, and complex overgrowth syndrome characterized by postnatal, asymmetric, disproportionate tissue hypertrophy, occurring in about 1:1,000,000 live births [9,10]. There are fewer than 200 cases reported in the medical literature [11]. Post-zygotic somatic mutations in the gene AKT1 have been linked to this mosaic disorder [12]. The clinical manifestations are highly variable and it affects males more than females, a ratio of 1.9:1 [9,10]. Knowledge of multiple clinical presentations is required for proper clinical diagnosis and management, especially for clinicians in resource-limited countries where genetic testing kits may be unavailable or unaffordable. Biesecker et al. proposed diagnostic criteria for individuals with features compatible with the syndrome [10,13]. The disproportionate overgrowth associated with PS may be so severe to the point that the individual is unrecognizable. Progressive bony overgrowth reduces joint mobility and further accentuates skeletal defects. Additional cardinal presentations include cerebriform connective tissue nevus, facial phenotypes, dysregulated increases in adipose tissue, and vascular malformations [10,13].

Our objective is to present the first documented rare case of an asymmetric overgrowth syndrome in the region and to highlight the complexity of the diagnosis and management of such cases.

## Case presentation

A seven-year-old African male was referred to the University of Nairobi Dental Hospital with a four-year history of progressive swelling of the left side of the face, left forearm, and toothache in the lower right and left quadrants for several months. He was brought to our facility by his mother, who was the informant. The painless swellings first appeared when he was three years old and progressively increased in size. The dental pain was nocturnal, aggravated by mastication, and easily localized to the offending teeth.

He is the younger of two children, both from non-consanguineous unions. The prenatal and perinatal periods were uneventful. He was born at term via spontaneous vertex delivery with a birth weight of 3.4 kg. There were no obvious dysmorphic features at birth. At the age of three years, the mother noticed progressive physical changes in her son's appearance and function. There was no familial history of similar physical features. There was no reported history of allergies to food or medication, hospital admission, or surgery. This was his index visit to the dental clinic. The systemic review demonstrated a history of slow progressive loss of power in the upper limbs (left > right), a long-standing pruritic rash, and multiple patchy areas of darkened skin. The family's social history revealed that he was from a single-parent, low-income household and had never been enrolled in school.

The patient walked in unaided and appeared stunted for age, withdrawn, and unkempt. He was able to follow simple instructions and respond mostly non-verbally by nodding his head or with single words. Left side facial hypertrophy and asymmetric overgrowth of the left upper limb were clearly observed. No obvious lower limb length discrepancy was apparent. His standing height was 98 cm and his weight was 17 kg. The skin was hyperkeratotic lichenified on the upper extremities, upper back, neck, and face. He had a generalized rash with multiple hyperpigmented cutaneous lesions, crusty flaking lesions on the scalp, a patch of alopecia on the left temporal region, and left palmar cerebriform soft tissue hypertrophy.

**Figure 1:**
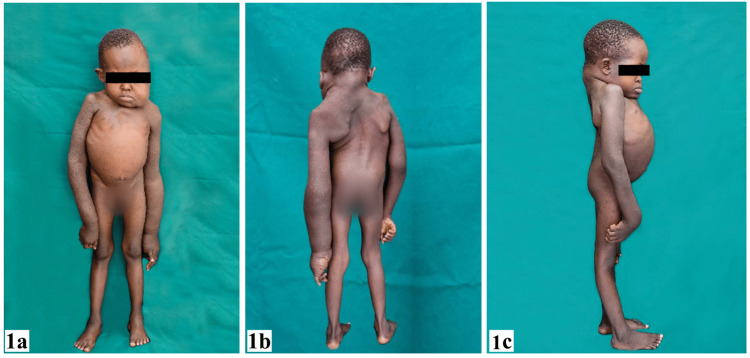
Clinical photographs. (a) Left hemihypertrophy of the face and left upper limb, and chest deformity; (b) and (c) cervicothoracic kyphoscoliosis (genitalia blurred out).

Musculoskeletal deformities were characterized by a short neck, pectus excavatum, cervicothoracic kyphoscoliosis, macrodactyly, and camptodactyly of the left fourth digit. These observations are illustrated in Figure 1.

He had reduced power in the upper limbs with a score of Medical Research Council (MRC) grade 4 on the right upper limb and MRC grade 3 on the left upper limb. Power in the lower limbs was normal.

**Figure 2:**
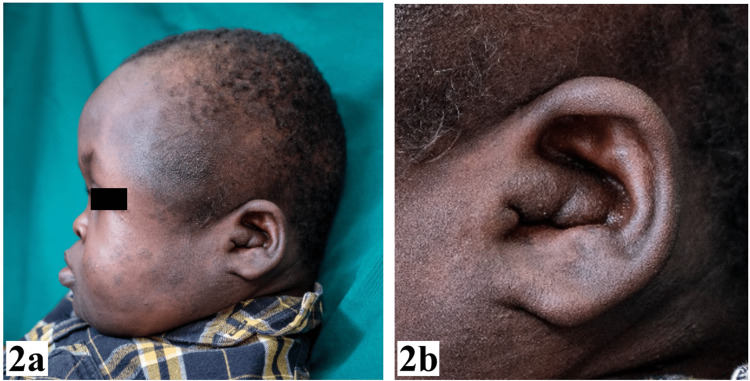
Extra-oral photographs. (a) Concave facial profile and (b) increased soft tissues of left ear.

The patient had a dolichocephalic head, a droopy facial appearance, a concave facial profile with mandibular prognathism, competent lips, and a flattened nasal bridge. Left facial hypertrophy of soft tissues and ptosis of the left eyelid with downward slanting of the palpebral fissure resulted in facial asymmetry. There was a wound on the left parietal scalp, and the right tympanic membrane was markedly inflamed. Additionally, there were increased soft tissues of the left outer ear, leading to severe stenosis of the left external auditory canal as illustrated in Figure 2.

**Figure 3:**
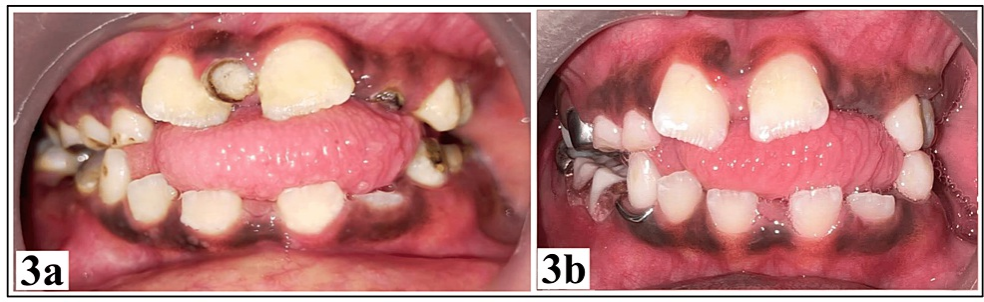
Intra-oral photographs. (a) Intra-oral presentation prior to oral rehabilitation; (b) intra-oral presentation after oral rehabilitation.

Intra-orally, he was in early mixed dentition and had generalized gross plaque accumulation. Asymmetric macroglossia was characterized by left-side hypertrophy, which resulted in an anterior and left-side posterior open bite. There were multiple grossly carious teeth, as illustrated in Figure 3.

Notably, he had attrition of the upper right teeth. The right first permanent molars were in Angle’s Class III molar relationship. However, the left first permanent molars were unerupted.

Relevant medical and dental specialist consultations were conducted to discuss the approach to the management of the patient. The diagnostic techniques used in the patient evaluation are presented in Table 1.

**Table 1:** Diagnostic tests applied in the case.

Investigation	Evaluation
Plain X-ray radiograph	Upper limbs
Computed tomography scans	Head, neck, chest, abdomen
Magnetic resonance imaging	Brain and thoracic spine
Orthopantomogram	Dental and maxillofacial region
Laboratory tests	Complete blood count, erythrocyte sedimentation rate, C-reactive protein, quantitative pro-calcitonin, renal function tests, liver function tests, 25 OH vitamin D3, D dimer assay genetic sequencing

**Figure 4:**
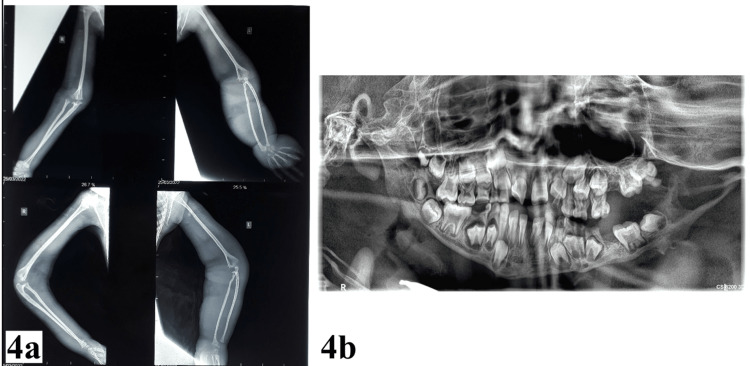
Plain radiographs. (a) X-ray of upper limbs showing dysplastic long bones and left macrodactyly; (b) there is agenesis of 38 and the morphology of the 34 is suggestive of germination.

The plain X-ray of his upper limbs showed dysplastic long bones with the increased prominence of the soft tissues on the left side. Dental radiographic findings included a delayed dental age estimated to be six to seven years, left side mandibular hypoplasia with coronoid and condylar elongation, irregular occlusal plane, grossly carious teeth, delayed root formation based on his chronological age, and taurodontism of the first permanent molars as illustrated in Figure 4.

**Figure 5:**
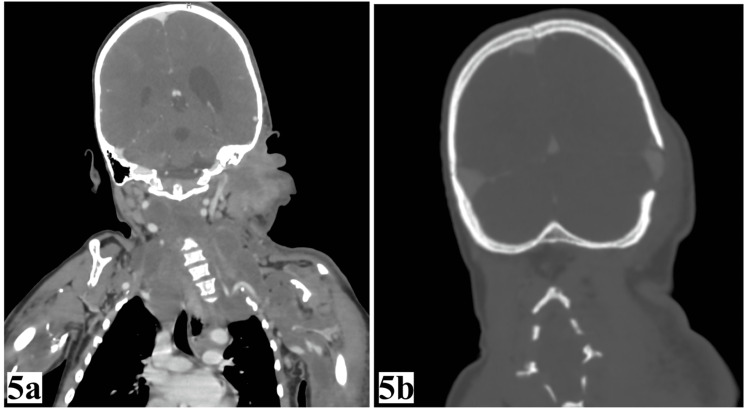
CT scan of the patient. (a) Cervicothoracic kyphoscoliosis; (b) diastasis of left lambdoid suture.

CT scans (contrast-enhanced) of the head, neck, chest, and abdomen found asymmetry of the cerebral hemispheres with a prominence of the left cerebral hemisphere and left lateral ventricle with diastasis of the left lambdoid suture while the right lambdoid suture was fused. There were extensive lesions suggestive of lymphangiomas of the left face, bilateral neck, and upper left limb, causing asymmetric hemihypertrophy. The spleen was slightly enlarged with a span of 14 cm. Significant S-shaped cervicothoracic kyphoscoliosis in the neck with concavity to the left in the cervical region and concave to the right in the upper thoracic region, as illustrated in Figure 5.

The larger Cobb angle is 40° and is in the thoracic region. The left hemithorax was slightly larger than the right.

**Figure 6:**
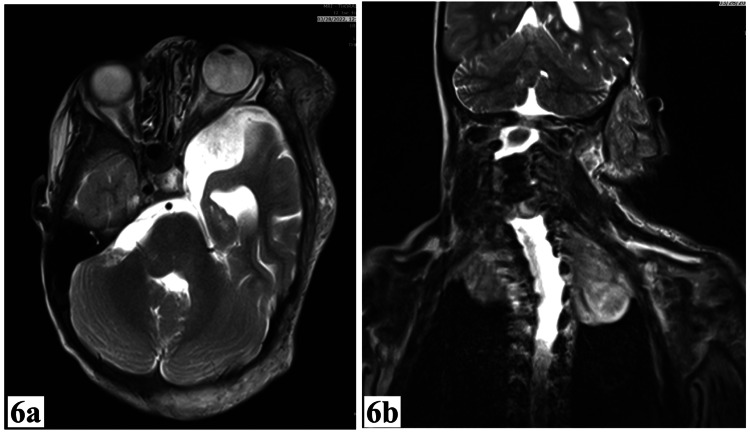
MRI of the patient. (a) Left eye proptosis and prominence of the left temporal lobe; (b) paravertebral masses are seen arising from the neural foramina and paravertebral regions.

MRI findings include prominence of the left temporal lobe; enlargement of the left lateral ventricle with dilatation of the CSF spaces of the ipsilateral middle cranial fossa and the lateral ventricle; and bilateral multiple neural foramina/paravertebral masses as illustrated in Figure 6.

The biochemistry, endocrinology, and D-dimer assay results were within normal ranges. Haematological findings were normal; however, the haemoglobin, mean corpuscular haematocrit, and mean corpuscular haematocrit concentration were borderline as presented in Table 2.

**Table 2:** Summary of the laboratory tests and results.

Test component	Findings	Normal range
Haemoglobin	11.4 g/dL	11.5–14.5 g/dL
Mean corpuscular haematocrit	23.9 pg	24–30 pg
Mean corpuscular haematocrit concentration	31.8 g/dL	32–36 g/dL

The paediatrician in attendance started him on iron 10 ml twice a day for four months, albendazole 20 ml stat dose, 4% chlorhexidine gluconate (hibiscrub) bath twice a day for ten days, mupirocin cream for the scalp wounds, and cefixime 200 mg once a day for ten days for the ear infection.

The patient underwent a custom-made Invitae® overgrowth syndrome, skeletal disorders, primary immunodeficiency, and a cancer predisposition gene panel. A total of 861 genes were sequenced and analyzed for deletions and duplications. However, a variation in the AKTI gene was not elicited in the sequencing.

Management of intra-oral findings involved comprehensive full mouth rehabilitation at the paediatric dental department, which involved oral prophylaxis and fluoride therapy, dental restorations on the carious teeth, and extractions of grossly carious teeth with space maintenance. The impact of his dental treatment was improved oral health with the elimination of dental pain and oral infections; improved masticatory function; and improved aesthetics. The patient is on follow-up by a paediatric haematologist, paediatric dentist, oral and maxillofacial surgeon, paediatric geneticist, and paediatric orthopaedic surgeon.

## Discussion

It is imperative to diagnose individuals with overgrowth syndromes for purposes of accurate genetic counselling, cancer surveillance, and overall prognosis [1].

**Figure 7:**
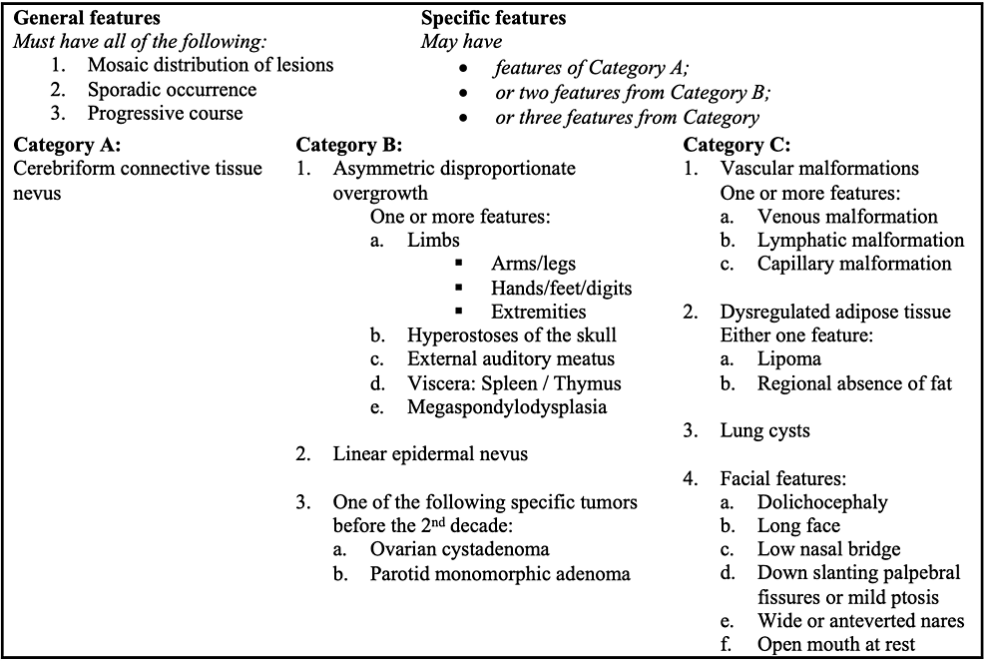
Diagnostic criteria of Proteus syndrome.

The patient presented with general and several specific features of PS [4,12]. These features included cerebriform connective tissue nevus and asymmetric disproportionate overgrowth of the left upper limb, left external auditory meatus, and spleen. Additionally, he had lymphangiomas, cystic lesions, lipomas, dolichocephaly, down-slanting palpebral fissures with ptosis, and a flattened nasal bridge. The patient’s clinical presentation fits the clinical criteria for diagnosis of PS, as an asymmetric overgrowth syndrome that is progressive and typically noted in childhood, as presented in Figure 7 [4,10,12,14,15].

Phenotypic presentations are induced by individual genes or different genes can produce one phenotype [16]. The genesis of PS is AKT1 gene variation (c.49G>A, p. Glu17Lys) [6]. The patient had a peripheral blood sequence analysis and deletion/duplication panel testing which did not elicit this variation. However, from the literature, diagnosis of PS from peripheral blood DNA analysis is achieved in only 5% of confirmed cases. A punch biopsy of the affected area, which was not approved by the parents, would offer a more definitive diagnosis [6].

The management of patients with overgrowth syndromes such as PS is challenging and requires a multidisciplinary team owing to the varied clinical presentation and risk factors [11]. Current treatment does not modify the disease progression and is, therefore, symptom-oriented [17]. Keppler-Noreuil et al. confirmed that individuals with PS have an increased risk of fatal thromboembolic events, though the underlying mechanism remains unknown [18,19]. This may occur due to vascular malformations resulting in stasis and an abnormal hypercoagulable panel characterized by deficiencies in anti-thrombin III and Protein C or S [18]. This necessitates the D-dimer test as a recommended screening tool in individuals with features compatible with PS [18]. Doppler ultrasound and a chest CT scan may identify deep venous thrombosis (DVT), pulmonary embolism (PE), and recurrent superficial venous thrombi. In patients with confirmed DVT or PE, acute anticoagulation therapy should be initiated.

Diagnostic imaging such as X-rays, MRIs, and CT scans is recommended for clinical care and management of individuals with overgrowth syndromes such as PS [19]. Scoliosis is a common skeletal manifestation of PS, as was evident in our case [20]. Scoliosis accompanied by severe distortion of the vertebrae, low bone density, reduced joint mobility, and neurological effects further complicates surgical planning [21]. The quality of life does not impose a surgical approach in all cases, as surgery in itself predisposes to thromboembolic events due to an added risk of prolonged immobilization [18]. However, surgical reconstruction may improve the physical appearance and emotional state of children with PS [21]. Mental retardation and seizures may be part of this syndrome, but the average intelligence usually remains normal [22,23]. Nevertheless, the patient did not manifest any history of seizures or mental retardation. In our case, full-mouth dental rehabilitation was possible using non-pharmacological behaviour modification techniques with moisture control performed using a rubber dam.

## Conclusions

Individuals with overgrowth syndromes, such as PS, suffer from a myriad of systemic manifestations and should be routinely monitored by a multidisciplinary team including a paediatric haematologist, paediatric dentist, oral and maxillofacial surgeon, and a paediatric orthopaedic surgeon. They should be treated for vascular problems and monitored for evidence of tumour development. Patients and parents should receive psychosocial support to encourage the societal integration of children with overgrowth syndromes. We recommend the inclusion of a child psychologist and a nutritionist in the care.
